# Retzius-sparing robot-assisted radical prostatectomy in a medium size oncological center holds adequate oncological and functional outcomes

**DOI:** 10.1007/s11701-022-01517-3

**Published:** 2023-01-12

**Authors:** Jorge Fonseca, Maria Francisca Moraes-Fontes, Jorge Rebola, Rui Lúcio, Miguel Almeida, Ciprian Muresan, Artur Palmas, Ana Gaivão, Celso Matos, Tiago Santos, Daniela Dias, Inês Sousa, Francisco Oliveira, Ricardo Ribeiro, Antonio Lopez-Beltran, Avelino Fraga

**Affiliations:** 1 Centro Clínico Champalimaud, Unidade de Próstata, Champalimaud Foundation, Av. Brasília, 1400-038 Lisbon, Portugal; 2 Centro Clínico Champalimaud, Unidade de Imuno-Oncologia, Champalimaud Foundation, Lisbon, Portugal; 3 Centro Clínico Champalimaud, Serviço de Imagiologia, Champalimaud Foundation, Lisbon, Portugal; 4 Centro Clínico Champalimaud, Unidade de Investigação Clínica, Champalimaud Foundation, Lisbon, Portugal; 5 Centro Clínico Champalimaud, Serviço de Medicina Nuclear, Champalimaud Foundation, Lisbon, Portugal; 6 Centro Clínico Champalimaud, Unidade de Anatomia Patológica, Champalimaud Foundation, Lisbon, Portugal; 7 Instituto de Ciências Biomédicas Abel Salazar, Universidade do Porto, Oporto, Portugal

**Keywords:** Prostate cancer, Retzius-sparing robot-assisted radical prostatectomy (RS-RARP), Positive surgical margin (PSM), Patient reported outcomes, International consultation on incontinence questionnaire-short form (ICIQ-SF), Sexual health inventory for men (SHIM), Expanded prostate index composite-26 (EPIC-26)

## Abstract

Retzius-sparing robot-assisted radical prostatectomy (RS-RARP) has emerged as a surgical option for patients with prostatic cancer in high-volume centers. The objective is to assess oncological and functional outcomes when implementing RS-RARP in a medium-volume center without previous experience of robotic surgery. This is a prospective observational single-center study. Patients operated between July 2017 and April 2020 were divided into two consecutive groups, A and B, each with 104 patients. The surgeons had prior experience in laparoscopic surgery and underwent robotic training. Positive surgical margin (PSM) status, urinary continence, and erectile function projected by Kaplan–Meier curves, together with patient reported quality of life outcomes at 12 months post-surgery were documented. Median patient age was 63 years (IQR = 59–67), overall PSM rate were 33%, 28% for pT2 disease. Pre-operative values showed no significant difference between both groups. The rate of urinary continence dropped from 81 to 78% (SE = 5.7) (Group A) and from 90 to 72% (SE = 6.3) (Group B) using the International Consultation on Incontinence Questionnaire-Short Form. Baseline sexual function was regained in 41% (Group A) and 47% (Group B) of patients. The median Expanded Prostate Index Composite-26 total score decreased from 86 to 82. These outcomes relate favorably to prior reports. There was a clinically significant decrease in median operative time in the successive groups with post-operative complications occurring in less than 2% of surgical procedures overall. A 12-month follow-up suggests that RS-RARP may be safely introduced in a medium-volume center without previous experience of robotic surgery.

**Supplementary Information:**

The online version contains supplementary material available at 10.1007/s11701-022-01517-3.

## Introduction

In developed countries, prostate cancer (PC) is the most frequently diagnosed cancer and the fifth leading cause of cancer death among men [1]. Radical prostatectomy is a treatment option with curative intent in clinically localized PC, in patients with a life expectancy exceeding 10 years [2]. The presence of a positive surgical margin (PSM) after surgery has prognostic significance for cancer recurrence [3] and is a surrogate marker of surgical quality [4].

Contemporary open radical prostatectomy (ORP) was described by Walsh et al. [5] in 1983, remaining largely unchallenged as the surgical technique of choice, until Guillonneau and Vallancien [6] described laparoscopic radical prostatectomy (LRP). Soon afterwards, in 2002, robot-assisted radical prostatectomy (RARP) was standardized [7], thus combining principles of ORP with concepts from LRP. Even though minimally invasive, whether laparoscopic or robotic, these techniques used the retropubic route, approaching the prostate anteriorly. In 2010, Galfano described the Bocciardi technique of Retzius-sparing RARP (RS-RARP), in which the prostate is approached posteriorly, through an incision in the parietal peritoneum, in the anterior aspect of the pouch of Douglas [8, 9]. Through this innovative surgical route, the prostate is immediately accessed, avoiding bladder dissection and damage to its supportive fascial structures. Over and above RARP, safer oncological outcomes together with a reduced number of adverse functional results have empowered RS-RARP as the technique of choice to be employed in high-volume centers with robotic facilities [10, 11].

The objective of this study is to determine the impact on patient outcomes from implementing RS-RARP in a medium-volume center such as ours, without previous experience in robotic surgery, by comparing two consecutive groups of patients. We hereby present prospectively collected real-life short-term oncological, functional and quality of life data in PC patients subjected to RS-RARP.

## Methods

### Patients and functional status evaluation

Our study included 208 patients that underwent RS-RARP with at least 1 year of follow-up, operated on by four surgeons, between July 2017 and April 2020. The cohort was divided into two consecutive  groups, each comprising 104 patients (Group A followed by Group B). Parameters were collected prospectively, including age and body mass index, preoperative oncological parameters such as prostate-specific antigen (PSA), highest International Society of Urological Pathology (ISUP) grade group at biopsy, and multi-parametric Magnetic Resonance Imaging (mpMRI)-based tumor clinical staging using Prostate Imaging-Reporting and Data System Version 2 (PI-RADS v2) [12].

Urinary incontinence was evaluated through pad use as well as the International Consultation on Incontinence Questionnaire-Short Form (ICIQ-SF) [13]. The Sexual Health Inventory for Men (SHIM) scores ≥ 17 or  ≥ 22 indicated mild or absent erectile dysfunction (ED), respectively, [14] regardless of whether potency-enhancing medication was used. Health-related quality of life (QoL) was evaluated through the Expanded Prostate Index Composite-26 (EPIC-26) questionnaire [15] and pre-established EPIC-26 Minimally Important Difference (MID) [16].

ICIQ-SF, pad-use and SHIM were documented every 3 months up to 12 months, whilst EPIC-26 was recorded at 12 months, postoperatively. All patients were considered incontinent and impotent in the immediate post-operative period. No patient required radiotherapy before continence or mild erectile function was achieved. QoL measures excluded any patient that required post-operative radiotherapy. PSA levels were recorded at 3 and 12 months post-operatively. All patients provided written informed consent for study inclusion, approved by the Institutional Ethics Committee (Approval 7.7.2017).

### Surgical technique

The surgical team comprised four surgeons who switched from ORP to LRP in 2014, with prior experience of 172 LRP procedures. The da Vinci Xi surgical system (Intuitive Surgical, Sunnyvale, CA, USA) became available in our hospital center from 2016. All surgeons were trained by Intuitive and the first eight patients, not included in this study, were proctored. RS-RARP was performed as described by Galfano [9]. A complete intrafascial procedure was performed for all patients who specifically demonstrated a desire for nerve preservation, with ISUP grade group < 4 disease on biopsy and mpMRI-based clinical stage < cT3. Unilateral or bilateral extrafascial dissection encompassing the neurovascular bundles was undertaken in patients with erectile dysfunction, high risk or locally advanced disease. Lymphadenectomy was performed in patients with a higher than 5% risk of nodal metastases, calculated according to the Memorial Sloan Kettering Cancer Center nomogram [17, 18]. Data related to the surgical procedure included operative time (skin-to-skin), estimated blood loss, blood transfusions, duration of hospital stay, bladder catheterization, and complications graded according to the Clavien–Dindo classification [19]. An indwelling Foley catheter was kept for at least 1 week. Performing approximately one hundred radical prostatectomies per year, our unit fits the definition of a medium volume center [20].

### Pathology examination

The same senior pathologist reviewed prostatic biopsies and surgical specimens. ISUP consensus and the American Joint Committee on Cancer 8th edition schemes were followed for grading and staging [21]. Pathological parameters included: prostate volume, tumor ISUP grade group, tumor stage, surgical margin status and length, and Gleason pattern of the PSM.

### Statistical analysis

Quantitative variables were reported as medians and interquartile range (IQR), and qualitative variables as counts or percentages for the full cohort as well as for each consecutive group. Results for functional outcomes were estimated using the Kaplan–Meier function, reported as percentage and standard error. All statistical analyses were performed using IBM® SPSS® Statistics version 27 for Windows, and a statistical significance level of 5% was defined.

## Results

### Pre-operative characterization

The overall median patient age was 63 years (IQR = 59–67) and most patients were slightly obese with a median BMI of 27. According to ICIQ-SF, the majority of patients (85%) did not suffer from urinary incontinence, only one patient reporting pad use. In contrast, the SHIM questionnaire yielded higher degrees of ED. Overall, 70% had erections sufficient for intercourse (SHIM ≥ 17) but only 41% of our patients had good erections (SHIM ≥ 22). Consistent EPIC-26 scores were found in the respective domains. There was homogeneity between the two consecutive groups as regards age, BMI, prostate size and PSA concentration. The ISUP grade ≥ 2 on prostate biopsy was slightly higher in group B patients but there were no significant differences in the MRI-based clinical stage. Functional and HRQoL evaluations were similar in both groups (Table 1).

**Table 1:** Preoperative features: demographics, disease stage, functional status and quality of life

Characteristic	Total	Group A	Group B
Patients evaluated, *n*	208	104	104
Age, years, median (IQR)	63 (59–67)	63 (59–67)	63 (59–68)
Body mass index, kg/m^2^, median (IQR)	27 (24–29)	26 (24–29)	27 (25–29)
Preoperative PSA, ng/ml, median (IQR)	7 (5–8)	7 (4 -6)	7 (5–9)
Prostate size, cm^3^, median (IQR)	43 (32–56)	43 (32–58)	43 (33–53)
Highest ISUP biopsy, *n* (%)	208 (100)	104 (100)	104 (100)
Grade 1	29 (14)	19 (18)	10 (10)
Grade 2	127 (61)	53 (51)	74 (71)
Grade 3	32 (15)	20 (19)	12 (11.5)
Grade 4	18 (9)	11 (11)	7 (7)
Grade 5	2 (1)	1 (1)	1 (1)
MRI-based T stage, *n* (%)	203 (98)	104 (100)	99 (95)^1^
T1c	20 (10)	8 (8)	12 (12)
T2a	57 (27)	32 (31)	25 (25)
T2b	25 (12)	13 (12)	12 (12)
T2c	41 (20)	19 (19)	22 (22)
T3a	53 (25)	28 (27)	25 (25)
T3b	7 (3)	4 (4)	3 (3)
Daily pad use, *n* (%)	197 (95)	97 (93)	100 (96)
No pad use	196 (99.5)	96 (99)	100 (100)
Pad use	1 (0.5)	1 (1)	0 (0)
ICIQ-SF, *n* (%)	198 (95)	97 (93)	101 (97)
ICIQ-SF = 0	168 (85)	78 (81)	90 (90)
ICIQ-SF > 0	30 (15)	19 (20)	11 (11)
SHIM, *n* (%)	195 (94)	95 (91)	100 (96)
No erectile dysfunction (SHIM ≥ 22), *n* (%)	80 (41)	40 (42)	40 (40)
Mild erectile dysfunction (17 ≤ SHIM ≤ 21), *n* (%)	56 (29)	29 (31)	27 (27)
Mild-Moderate erectile dysfunction (12 ≤ SHIM ≤ 16), *n* (%)	29 (15)	8 (8)	21 (21)
Moderate erectile dysfunction (8 ≤ SHIM ≤ 11), *n* (%)	11 (5)	7 (7)	4 (4)
Severe erectile dysfunction (SHIM ≤ 7), *n* (%)	19 (10)	11 (11)	8 (8)
EPIC-26 score, *n*	197	97	100
Median (IQR)	86 (79–95)	86 (78–95)	86 (79–94)

Legend: Shown are the total number of patients evaluated for each parent variable, subsequently followed by the corresponding number and percentage of the sub-variable. *PSA:* prostate-specific antigen; *ISUP:* International Society of Urological Pathology; *MRI:* Magnetic Resonance Imaging; ^1^: MRI stage not performed in 5 patients in group B due to contra-indication (*n*  = 1) and poor image resolution (*n* = 4); ICIQ-*SF:* International Consultation on Incontinence Questionnaire-Short Form; *SHIM:* Sexual Health Inventory for Men; *EPIC*-26: Expanded Prostate Index Composite-26.

### Surgical procedure

Both consecutive groups remained comparable with respect to the number of patients subjected to extended pelvic lymph node dissection (46 and 42%) and the median number of lymph nodes removed (*n* = 21). There was also a similar number of patients in whom bilateral and unilateral neurovascular sparing (circa 80%) and bladder neck preservation (over 90%) was performed, with equivalent intraoperative blood loss (200 ml), post-operative complications (0 to 2%), median length of hospital stay (2 to 3 days) and duration of catheterization (8 to 9 days). Whereas the median intraoperative time remained similar as regards radical prostatectomy alone (215 to 220 min), there was a clinical significant reduction from 349 to 300 min with respect to radical prostatectomy accompanied by lymph node dissection, from Group A to Group B (Table 2).

**Table 2:** Retzius sparing prostatectomy: intra- and postoperative results

Characteristic	Total	Group A	Group B
Recorded lymph node dissection status for all patients, *n* (%)	208 (100)	104 (100)	104 (100)
Lymphadenectomy	92 (44)	48 (46)	44 (42)
No lymphadenectomy	116 (56)	56 (54)	60 (58)
Patients with recorded nerve sparing status, *n* (%)	167 (80)	76 (73)	91 (87)
No nerve sparing	34 (20)	13 (17)	21 (23)
Unilateral	57 (34)	28 (37)	29 (32)
Bilateral	76 (46)	35 (46)	41 (45)
Patients with recorded bladder neck dissection status, *n* (%)	145 (70)	65 (62)	80 (77)
Bladder neck preserved	134 (93)	60 (92)	74 (92)
Bladder neck not preserved	11 (7)	5 (8)	6 (8)
Recorded operative time, n patients in whom recorded (%)	203 (98)	104 (100)	99 (95)
Radical prostatectomy, minutes, median (IQR)	219 (188–254)	220 (186–262)	215 (188–251)
Radical prostatectomy plus lymph node dissection, minutes, median (IQR)	320 (280–369)	349 (307–405)	300 (270–330)
Lymph nodes removed, median (IQR)	21 (17–24)	21 (17–24)	21 (17–24)
Intraoperative blood loss, ml, median (IQR)	200 (150–300)	200 (150–300)	200 (150–300)
Intraoperative transfusion rate, *n*	0	0	0
Intraoperative complications, *n*	0	0	0
Postoperative complications (Clavien-Dindo > II):			
Surgery for hemostasis, *n* (%)	2 (1)	2 (2)	0
Lymphocele drainage, *n* (%)	2 (1)	0	2 (2)
Length of hospital stay, days, median (IQR)	2 (2–3)	3 (2–3)	2 (2–3)
Duration of catheterization, days, median (IQR)	8 (8–9)	8 (8–9)	9 (8–9)

Legend: Shown are the total number of patients evaluated for each variable, subsequently followed by the corresponding number and percentage of the sub-variable with respect to the parent variable.

### Oncological outcomes

Postoperatively, the majority of patients had a PC staged as pT2, 70% in Group A and 60% in Group B. The overall PSM for all pathological T stages (pT) was described in 33% of patients (Table 3). A PSM for pT2 patients was reported in 29% and 27% of Groups A and B, respectively. There was a lower proportion of patients with local advanced tumors (pT3) in Group A (30%) than in Group B (40%) and accordingly, for this stage, the PSM rate was 39% and 44%, respectively. Both were similar in terms of the degree of extensive disease and Gleason score of the surgical margin, pelvic lymph node involvement, biochemical recurrence inferior to 10% at 12 months post-operatively, and the proportion that required adjuvant and salvage treatments (25%).

**Table 3:** Postoperative pathological evaluation, oncological outcomes and quality of life assessment

Characteristic	Total	Group A	Group B
Patients evaluated, *n*	208	104	104
Pathological T stage (pT)			
pT2, *n* (%)	136 (65)	73 (70)	63 (60)
pT3a, *n* (%)	56 (27)	23 (22)	33 (32)
pT3b, *n* (%)	16 (8)	8 (8)	8 (8)
Positive margin according to pT			
*n* (% pT2)	38 (28)	21 (29)	17 (27)
*n* (% pT3a)	23 (41)	7 (30)	16 (48)
*n* (% pT3b)	7 (44)	5 (63)	2 (25)
Surgical margin			
Overall negative, *n* (%)	140 (67)	71 (68)	69 (66)
Overall positive, *n* (%)	68 (33)	33 (32)	35 (34)
Pattern surgical margin (% of positive margin)			
Focal (≤ 3 mm), *n* (%)	29 (43)	14 (44)^1^	15 (43)
Extensive (> 3 mm), *n* (%)	39 (57)	18 (56)^1^	20 (57)
Gleason score for the surgical margin (% of positive margin)			
3 + 3, *n* (%)	16 (24)	12 (36)	4 (12)
3 + 4, *n* (%)	34 (51)	14 (42)	20 (59)
4 + 3, *n* (%)	12 (18)	4 (12)	9 (26)
4 + 4, *n* (%)	5 (7)	3 (9)	2 (6)
*N* stage			
pNx, *n* (%)	116 (56)	56 (54)	60 (58)
pN0, *n* (%)	73 (35)	40 (39)	33 (32)
pN1, *n* (%)	19 (9)	8 (8)	11 (11)
PSA persistence and recurrence			
PSA ≥ 0.2 ng/ml at 3 months, *n* (%)	15 (7)	8 (8)	7 (7)
PSA ≥ 0.2 ng/ml at 12 months, *n* (%)	18 (9)	8 (8)^1^	10 (10)^1^
Adjuvant and salvage treatments	52 (25)	25 (24)	27 (26)
Androgen deprivation therapy, *n* (%)	2 (1)	0	2 (7)
Radiotherapy, *n* (%)	38 (8)	19 (18)	19 (18)
Androgen deprivation therapy + radiotherapy, *n* (%)	12 (6)	6 (6)	6 (6)
EPIC-26 score, *n*	136	68	68
Median (IQR)	82 (74–89)	82 (74–89)	82 (74–89)

Legend: *ISUP:* International Society of Urological Pathology; *PSM:* Positive Surgical Margin; *PSA:* prostate specific antigen; *ADT:* androgen deprivation therapy; *RT:* radiotherapy; *EPIC*-26: Expanded Prostate Index Composite-26. ^1^: PSA already ≥ 0.2 ng/ml at 3 months for 2 patients in Group A and 3 patients in Group B.

### Post-operative functional recovery

**Fig. 1:**
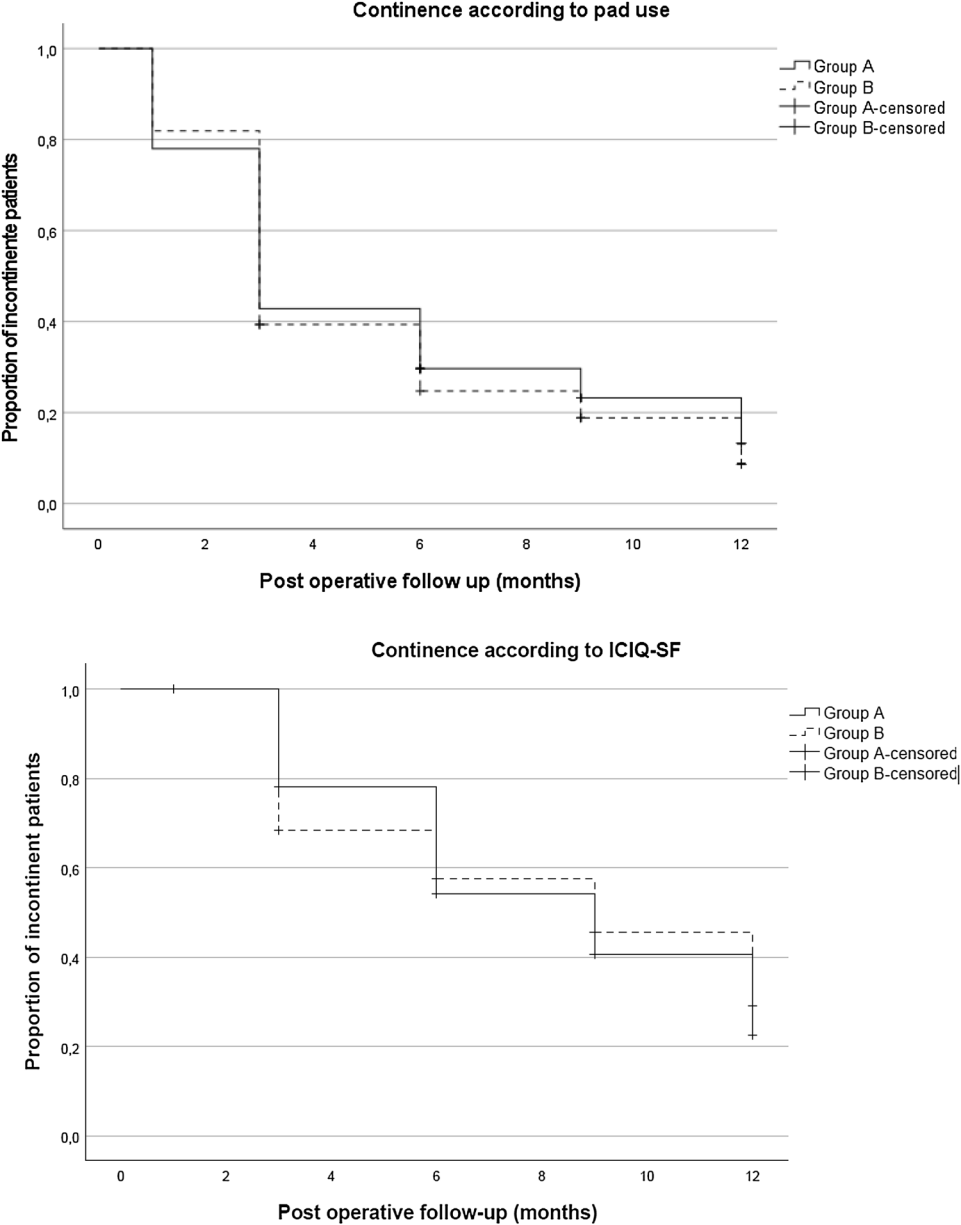
Kaplan–Meier curves show the proportion of continent patients according to pad use and ICIQ-SF recorded at 3, 6, 9, and 12 months postoperatively. All patients are considered incontinent in the immediate post-operative period. Continence is considered when there is no pad use or the ICIQ-SF score is equal to zero. Patients are censored when continence is reached.

**Fig. 2:**
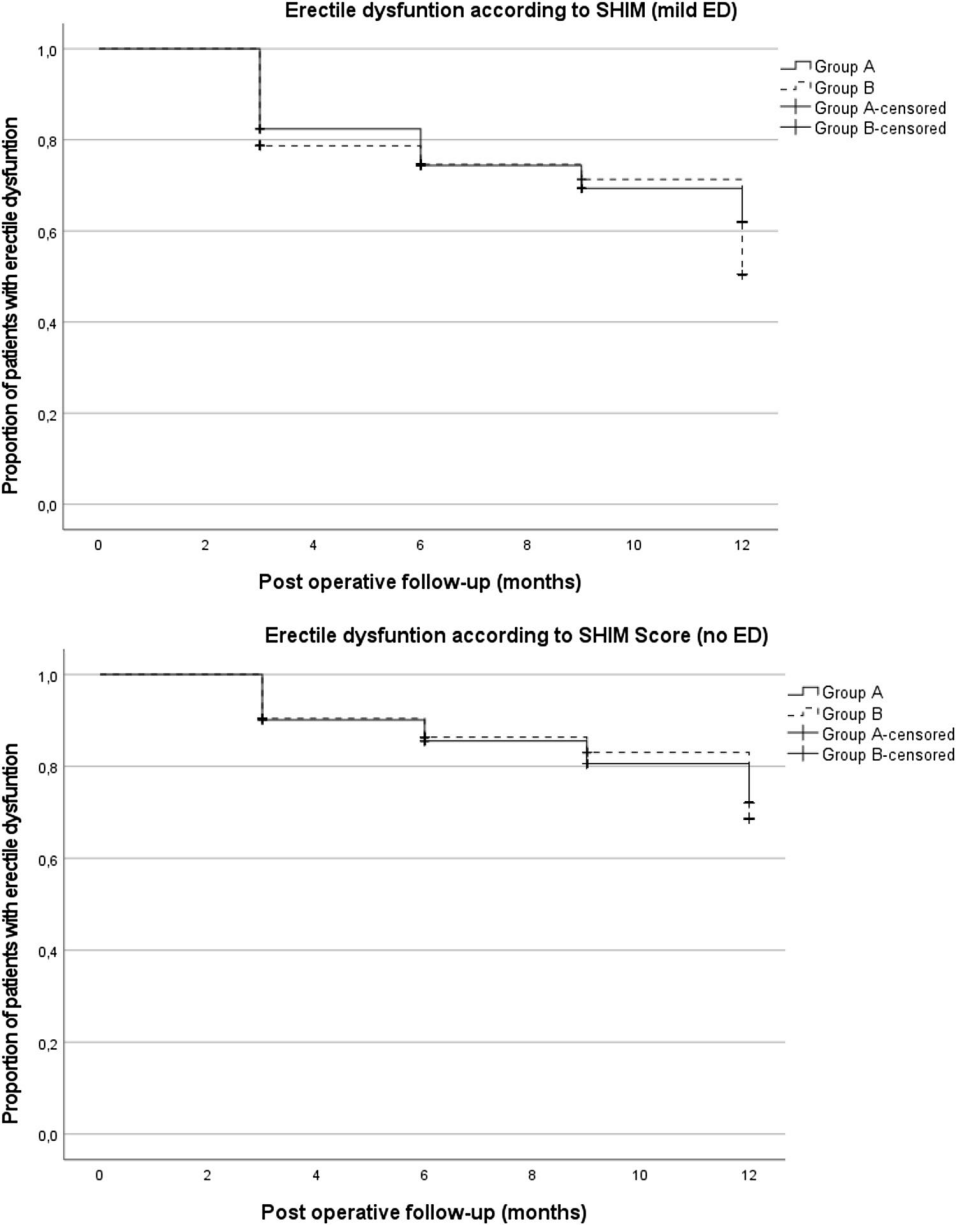
Kaplan–Meier curves show the proportion of patients with erectile function measured through the SHIM questionnaire, recorded at 3, 6, 9, and 12 months postoperatively. Severe erectile dysfunction was considered in all patients in the immediate post-operative period. A SHIM score ≥ 17 indicates mild dysfunction, yet sufficient for sexual intercourse (mild ED); a SHIM score ≥ 22 indicates no dysfunction (no ED). Patients are censored when the respective category of sexual function is reached.

Serial longitudinal evaluations obtained from group A (*n* = 91) and group B (n = 94) revealed that at 12 months, the frequency of continent patients steadily increased to 87% (SE = 4.1) and 92% (SE = 3.3) considering pad use,  and to 78% (SE = 5.7) and 72% (SE = 6.3) considering ICIQ-SF, respectively. Of note, 50% of the patients were continent by 3 months when considering pad use, a value only achieved at 9 months through the ICIQ-SF questionnaire (Fig. 1). Erectile dysfunction was much more frequent than urinary incontinence. Nevertheless, Kaplan–Meier curves projected a steady improvement in erectile function. At 12 months, good erections (SHIM ≥ 22) and erections sufficient for intercourse (SHIM ≥ 17) were reported by 29% (SE = 5.2) and 39% (SE = 5.6) in group A and 33% (SE = 6.3) and 50% (SE = 6.5) in group B, respectively (Fig. 2). None of the included patients required radiotherapy before attaining continence or a SHIM value ≥ 17 or ≥ 22.

**Fig. 3:**
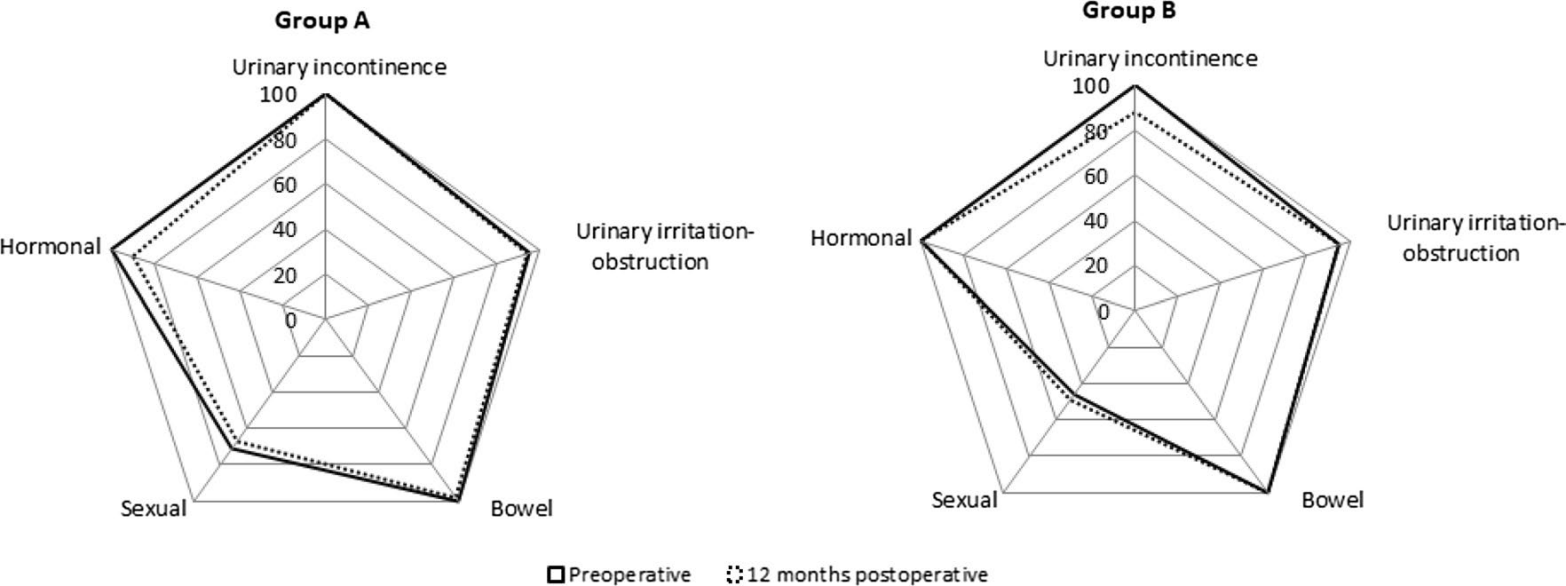
Shown are radar charts for EPIC-26 scores in Groups A (*n* = 68) and B (*n* = 68) pre-operatively and at 12 months follow-up.In this sub-group, compared with preoperative scores, the hormonal domain alone decreased in Group A, reaching MID significance. On the offset, the urinary incontinence domain alone was poorer in Group B, reaching MID.

EPIC domains were only evaluated in 68/104 patients in each group (Fig. 3). Exclusions from continence and erectile function analysis were due to loss early in follow-up (13 in group A and 10 in group B), radiotherapy treatment (13 in group A and 19 in group B) and absence at the 12 month assessment (10 in group A and 7 in group B). EPIC-26 was significantly associated with pad use and ICIQ-SF (*p* < 0.001 in both – *t* test), the latter exerting a superior effect size (Cohen’s d = 1.056 vs 0.973).

### Sequential functional outcome comparisons to pre-operative measures

In our series, at 1 year, when compared to pre-operative values, the estimated rate of urinary continence dropped from 99% to 86% (SE = 4.1) (A) and 100% to 92% (SE = 3.3) (B) when obtained through pad use, and from 81% to 78% (SE = 5.7) (A) and 90% to 72% (SE = 6.3) (B) through the ICIQ-SF questionnaire. There was no significant difference between both groups. A sub-group analysis at 12 months was slightly different from the projected continence rates provided by Kaplan–Meier curves after surgery. From the group of pre-surgical continent patients (ICIQ-SF = 0), information was only available from 74% (*n* = 57) and 66% (*n* = 60) patients from groups A and B, respectively. In group A, the majority (61%) remained continent (20 patients having been excluded from the analysis at 12 months). Conversely, in Group B, four patients who were incontinent pre-operatively became continent at 12 months (Supplementary Table I).

Regarding erectile dysfunction, when compared to pre-operative values, at 12 months, patients with erection sufficient for intercourse (SHIM ≥ 17) decreased from 73 to 39% (SE = 5.6) (A) and from 67 to 50% (SE = 6.5) (B); the number of potent patients (SHIM ≥ 22) dropped from 43 to 29% (SE = 5.2) (A) and from 40 to 33% (SE = 6.3) (B). Once again, the sub-group analysis at 12 months revealed that information was only available for 74% (*n* = 51) and 67% (*n* = 45) of patients in groups A and B, respectively. In the same group order, potency sufficient for sexual intercourse (SHIM ≥ 17) was effectively regained in 41 and 47% (Supplementary Table II).

Overall, the median EPIC-26 score decreased from 86 preoperatively to 82, at 1 year. Taking into consideration the full patient cohort pre-operatively there seemed to be a negative impact in sexual function (Supplementary Fig. 1). However, data was only available for 68/104 patients in each group. Demographic and clinical sub-group analysis for these EPIC-evaluated patients at 12 months showed homogeneity with the original group (Supplementary Table III).

## Discussion

As judged by an absence of intraoperative complications and a very low rate of post-operative complications that remained similar, at a value of 2%, in both successive groups of patients, RS-RARP was safely implemented in our medium volume center, in line with recent work [8, 22]. This was accomplished in our patients, despite reservations posed by the known association between high-volume surgeons and centers with lower complication rates and better outcomes [23, 24]. From the first to the second group, there was a clinically significant decrease in median operative time of 49 minutes.

Encountered during the implementation stage of an innovative surgical technique in a medium-volume center, our limitations namely, the small sample size, the 12-month follow-up and drop-out rate as well as the single-center nature of our study, prevented statistical inference for most parameters and evaluation of long-term clinical benefit. Nevertheless, PSM status remains a robust measure of both surgical quality and early adverse oncological outcomes, clearly influenced by the pathological T stage of the tumor and surgical experience, from which we can acknowledge adequate and safe outcomes from RS-RARP introduction in our center. In a recent series of 210 RARPs performed by four consultant surgeons with background experience of 374 LRPs, tumor stage was pT3 in 30% of the patients and the PSM rate was 29.5% [25]. This was the nearest we could find to our study, comparably comprising a similar frequency of pT3 tumors (Group A = 30%; Group B = 40%) with an overall PSM rate of 33% (Group A = 32%; Group B = 34%). Community-based centers also provide baseline data to judge whether our results reflect safe short-term oncological outcomes. At a population audit setting, several studies report PSM rates of 33% [26] and 24% [27] for pT2 disease, standing similar to our overall pT2 PSM rate of 28% (Group A = 29%; Group B = 27%). Polarized results from highly experienced centers, despite a similar frequency of pT3 tumors (24 to 33%), lower the PSM rate to as much as 19% [28] to 21% [29], respectively. Alike a recent study, [30] the biochemical free survival determined by PSA concentration was similar in Groups A and B (92 and 90%),

Given the high survival rates of PC patients, functional results are important clinical endpoints. We chose to collect data regarding safety-pad use per day and ICIQ-SF, bearing in mind that the definition of urinary continence is heterogeneous, and methods used to collect and report data may influence the rate of post-operative incontinence [31]. In our study, Kaplan–Meier curves project a steady rise in post-operative continence rates post-operatively. Considering pad use, less than 10% of the patients are incontinent at 12 months post-operatively. This is in line with a similar study employing RS-RARP [30] and an improvement when compared to an extensive review reporting urinary continence recovery after conventional RARP, where the 12-months incontinence rate ranged from 4 to 31%, with a mean value of 16% using a “no pad” definition [32].

Measured using the ICIQ-SF, there was a similar rise in the frequency of post-operative incontinence in both groups. The preoperative urinary continence rate for our two consecutive groups A and B was 81% and 90%, decreasing to 77% and 72%, respectively, at 12 months post-operatively. Once again, our patients demonstrate major progress in continence rates when compared to recent conventional RARP results where only 31% of the patients returned to an ICIQ-SF score of zero at 1 year after robotic surgery [33]. ICIQ-SF information as regards the actual number of pre-surgical continent patients that remain continent at 12 months was only available for less than two-thirds of the patients. Notwithstanding a higher loss of continence in group B than in Group A at 12 months, four pre-operatively incontinent patients became continent by 12 months in Group B.

Concerning erectile function, in both group of patients, there was a steady recovery of potency and erections sufficient for intercourse as follow-up continued up to 12 months. Considering patients with erections sufficient for intercourse pre-operatively (SHIM ≥ 17), 41% (Group A) and 47% (Group B) had regained baseline sexual function at 12 months, in line with a recent report [34].

The ICIQ-SF was more sensitive (larger effect size) than daily pad count at capturing the impact of incontinence on HRQoL measured by the EPIC-26. The impact on HRQoL, evaluated with EPIC-26 at 1 year of surgery was similar in both groups with a reduction in the urinary incontinence domain of 10%, meeting the lower value of the MID range, in line with the slightly higher increase in post-operative urinary incontinence in Group B.

## Conclusion

We present results from 208 PC patients submitted to RS-RARP and analyzed in two consecutive homogenous groups. There was a significant reduction in operating times from the first group to the next. The frequencies of PSM, urinary continence recovery and biochemical recurrence at 12 months were similar in both groups, in line with published RS-RARP series and improved with respect to RARP results. Overall patient QoL was maintained. Our results suggest that RS-RARP may be safely introduced in a medium-volume center, with multisurgeon implementation without previous experience in robotic surgery, hopefully contributing to the generalization of the RS-RARP in community centers in a “real world” setting.

## Supplementary Information

Below is the link to the electronic supplementary material.

Supplementary file1 (DOCX 117 KB)

## Data Availability

The datasets generated during and/or analysed during the current study are available from the corresponding author on reasonable request.
