# Supplementary material for: Retzius-sparing robot-assisted radical prostatectomy in a medium size oncological center holds adequate oncological and functional outcomes
Source: J Robot Surg. 2023 Jan 12;17(3):1133–42. doi: 10.1007/s11701-022-01517-3 (PMC10209308; doi:10.1007/s11701-022-01517-3)

Title: Supplementary Table I. Evaluation of pre-operative patients according to continence status at 12 months post-operatively

| Pre-operative continence | Group A | Group B |
| --- | --- | --- |
| a) Evaluated patients pre-operative, n | 96 | 101 |
| b) Pre-operative continent (ICIQ-SF = 0), n (% of a) | 77 (80) | 91 (90) |
| c) Number evaluated patients postoperative (12 months), n (% of b) | 57 (74) | 60 (66) |
| ICIQ-SF = 0, n (% of c) | 35 (61) | 23 (38) |
| ICIQ-SF>0, n (% of c) | 22 (39) | 37 (62) |
| Exclusions, n | 20 | 31 |
| After Radiotherapy, n | 7 | 15 |
| Lost to follow-up<12 months, n | 3 | 8 |
| Lost to follow-up at 12 months, n | 10 | 8 |
| d) Pre-operative incontinent - ICIQ-SF>0, n (% of a) | 19 (20) | 10 (10) |
| e) Number evaluated patients postoperative (12 months), n (% of d) | 8 (42) | 6 (60) |
| ICIQ-SF>0, n (%) | 8 (100) | 2 (33) |
| ICIQ-SF = 0, n (%) | 0 (0) | 4 (67) |
| Exclusions, n | 11 | 4 |
| After Radiotherapy, n | 3 | 1 |
| Lost to follow-up<12 months, n | 6 | 2 |
| Lost to follow-up at 12 months, n | 2 | 1 |

Title: Supplementary Table II. Evaluation of pre-operative patients according to erectile function status at 12 months post-operatively

| Pre-operative erectile function | Group A | Group B |
| --- | --- | --- |
| a) Evaluated patients pre-operative, n | 95 | 100 |
| b) Pre-operative SHIM ≥ 17, n (% of a) | 69 (73) | 67 (67) |
| c) Number evaluated patients postoperative (12 months), n (% of b) | 51 (74) | 45 (67) |
| SHIM ≥ 17, n (%) | 21 (41) | 21 (47) |
| SHIM ≤ 16, n (%) | 30 (59) | 24 (53) |
| Exclusions, n | 18 | 22 |
| After Radiotherapy, n | 7 | 10 |
| Lost to follow-up<12 months, n | 5 | 6 |
| Lost to follow-up at 12 months, n | 6 | 6 |
| d) Pre-operative SHIM ≥ 22, n (% of a) | 40 (42) | 40 (40) |
| e) Number evaluated patients postoperative (12 months), n (% of d) | 31 (78) | 26 (65) |
| SHIM ≥ 22, n (%) | 12 (39) | 12 (46) |
| SHIM ≤ 21, n (%) | 19 (61) | 14 (54) |
| Exclusions, n | 9 | 14 |
| After Radiotherapy, n | 6 | 8 |
| Lost to follow-up<12months, n | 1 | 2 |
| Lost to follow-up at 12 months, n | 2 | 4 |

Title: Supplementary Table III. Demographic, clinical, operative and pathologic characteristics of EPIC-26 evaluated patients with respect to the overall number of patients in each group

|  | Group A | | Group B | |
| --- | --- | --- | --- | --- |
| Characteristic | Initial Group N=104 | Assessed for functional outcome at 12 months N=68 | Initial Group N=104 | Assessed for functional outcome at 12 months N=68 |
| Age, years, median (IQR) | 63 (59-67) | 63 (59-67) | 63 (59-68) | 64 (59-68) |
| Body mass index, kg/m^2^, median (IQR) | 26 (24-29) | 26 (24-28) | 27 (25-29) | 28 (25-29) |
| Preoperative PSA, ng/ml, median (IQR) | 7 (4 -6) | 7 (5-8) | 7 (5-9) | 6 (5-8) |
| Prostate size, cm^3^, median (IQR) | 43 (32-58) | 46 (33-57) | 43 (33-53) | 45 (36-55) |
| Highest ISUP grade at biopsy for all patients, n (%) | 104 (100) |  | 104 (100) |  |
| Grade 1 | 19 (18) | 15 (22) | 10 (10) | 7 (10) |
| Grade 2 | 53 (51) | 38 (56) | 74 (71) | 53 (78) |
| Grade 3 | 20 (19) | 8 (12) | 12 (11.5) | 4 (6) |
| Grade 4 | 11 (11) | 6 (9) | 7 (7) | 4 (6) |
| Grade 5 | 1 (1) | 1 (1) | 1 (1) | 0 (0) |
|  |  |  |  |  |
| MRI-based T stage, n (%) | 104 (100) | 68 (100) | 99 (95) | 65 (96) |
| T1c | 8 (8) | 5 (7) | 12 (12) | 9 (14) |
| T2a | 32 (31) | 24 (35) | 25 (25) | 18 (28) |
| T2b | 13 (12) | 8 (12) | 12 (12) | 10 (15) |
| T2c | 19 (19) | 17 (25) | 22 (22) | 15 (23) |
| T3a | 28 (27) | 2 (3) | 25 (25) | 12 (18) |
| T3b | 4 (4) |  | 3 (3) | 1 (2) |
|  |  |  |  |  |
| Lymphadenectomy | 48 (46) | 28 (41) | 44 (42) | 22 (32) |
|  |  |  |  |  |
| No Nerve sparing | 13 (17) | 28 (41)^1^ | 21 (23) | 10 (17) ^1^ |
|  |  |  |  |  |
| Bladder neck preserved | 60 (92) | 8 (16)^2^ | 74 (92) | 49 (92) ^2^ |
|  |  |  |  |  |
| Histological evaluation: |  |  |  |  |
| T stage, n (%) |  |  |  |  |
| pT2 | 73 (70) | 54 (79) | 63 (60) | 54 (79) |
| pT3a | 23 (22) | 11 (16) | 33 (32) | 13 (19) |
| pT3b | 8 (8) | 3 (4) | 8 (8) | 1 (1) |
|  |  |  |  |  |
| Surgical margin: |  |  |  |  |
| Overall negative, n (%) | 71 (68) | 51 (75) | 69 (66) | 5 (79) |
| Overall positive, n (%) | 33 (32) | 17 (25) | 35 (34) | 14 (21) |
| Positive margin according pathological T stage (pT): |  |  |  |  |
| pT2, n (%) | 21 (29) | 14 (26) | 17 (27) | 12 (22) |
| pT3a, n (%) | 7 (30) | 2 (18) | 16 (48) | 2 (15) |
| pT3b, n (%) | 5 (63) | 1 (33) | 2 (25) | 0 (0) |
|  |  |  |  |  |
| N stage: |  |  |  |  |
| pNx, *n* (%) | 56 (54) | 40 (59) | 60 (58) | 46 (68) |
| pN0, *n* (%) | 40 (39) | 24 (35) | 33 (32) | 21 (31) |
| pN1, *n* (%) | 8 (8) | 4 (6) | 11 (11) | 1 (1) |

Legend: ^1^=Information recorded for 49 patients in group A and 60 patients in group B; ^2^= Information recorded for 43 patients in group A and 53 patients in group B.

Title: Supplementary Figure 1. Comparison between EPIC-26 scores in Groups A and B pre-operatively (n=104) and post-operatively at 12 months follow-up (n=68).


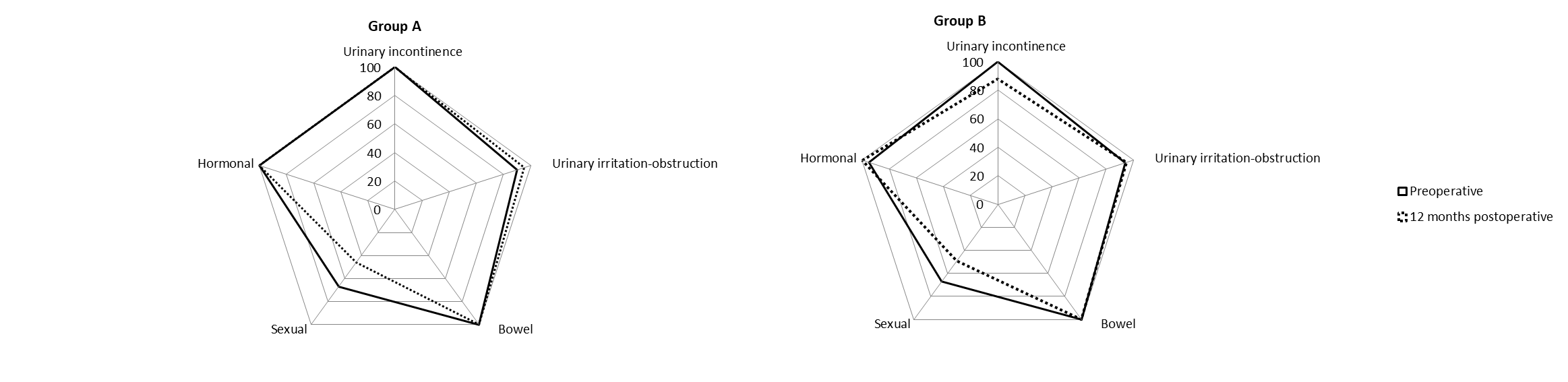


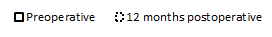

Supplement: Supplementary file 1 — Supplementary file1 (DOCX 117 KB) [file 11701_2022_1517_MOESM1_ESM.docx]
